# Malaria transmission in two localities in north-western Argentina

**DOI:** 10.1186/1475-2875-8-18

**Published:** 2009-01-19

**Authors:** María J Dantur Juri, Mario Zaidenberg, Guillermo L Claps, Mirta Santana, Walter R Almirón

**Affiliations:** 1 Instituto Superior de Entomología "Dr. Abraham Willink", Facultad de Ciencias, Naturales e Instituto Miguel Lillo, Universidad Nacional de Tucumán, Miguel Lillo 205, CP 4000, Tucumán, Argentina; 2 Coordinación Nacional de Control de Vectores, Ministerio de Salud de la Nación, Güemes 125, Piso 1, CP 4400, Salta, Argentina; 3 Cátedra de Bioestadística, Facultad de Medicina, Universidad Nacional de Tucumán, Lamadrid 875, CP 4000, Tucumán, Argentina; 4 Centro de Investigaciones Entomológicas de Córdoba, Facultad de Ciencias Físicas, Exactas y Naturales, Universidad Nacional de Córdoba, Av. Vélez Sarfield 1611, CP 5016, Córdoba, Argentina

## Abstract

**Background:**

Malaria is one of the most important tropical diseases that affects people globally. The influence of environmental conditions in the patterns of temporal distribution of malaria vectors and the disease has been studied in different countries. In the present study, ecological aspects of the malaria vector *Anopheles *(*Anopheles*) *pseudopunctipennis *and their relationship with climatic variables, as well as the seasonality of malaria cases, were studied in two localities, El Oculto and Aguas Blancas, in north-western Argentina.

**Methods:**

The fluctuation of *An. pseudopunctipennis *and the malaria cases distribution was analysed with Random Effect Poisson Regression. This analysis takes into account the effect of each climatic variable on the abundance of both vector and malaria cases, giving as results predicted values named Incidence Rate Radio.

**Results:**

The number of specimens collected in El Oculto and Aguas Blancas was 4224 (88.07%) and 572 (11.93%), respectively. In El Oculto no marked seasonality was found, different from Aguas Blancas, where high abundance was detected at the end of spring and the beginning of summer. The maximum mean temperature affected the *An. pseudopunctipennis *fluctuation in El Oculto and Aguas Blancas. When considering the relationship between the number of malaria cases and the climatic variables in El Oculto, maximum mean temperature and accumulated rainfall were significant, in contrast with Aguas Blancas, where mean temperature and humidity showed a closer relationship to the fluctuation in the disease.

**Conclusion:**

The temporal distribution patterns of *An. pseudopunctipennis *vary in both localities, but spring appears as the season with better conditions for mosquito development. Maximum mean temperature was the most important variable in both localities. Malaria cases were influenced by the maximum mean temperature in El Oculto, while the mean temperature and humidity were significant in Aguas Blancas. In Aguas Blancas peaks of mosquito abundance and three months later, peaks of malaria cases were observed. The study reported here will help to increase knowledge about not only vectors and malaria seasonality but also their relationships with the climatic variables that influence their appearances and abundances.

## Background

Malaria, one of the most important human parasitic diseases, seemed to be under control in the 1950s. During the last 30 years, however, there has been a recrudescence of malaria in many regions of the world and currently it is the main vector-borne disease in developing countries. In Argentina, malaria cases decreased towards the end of 1980 and increased again in 2000 by almost 100%; 80% of these cases were recorded as imported [1]. The provinces most affected by the disease were Salta and Jujuy, located on the border between Argentina and Bolivia, where human migration and the lack of vector control are among the factors hypothesized to favour malaria transmission.

The development of the disease in north-western Argentina has always been influenced by the topography, climate and phytogeography [2]. Thus, the so-called "mountainous malaria area" can be clearly defined both in the northern and in the central Argentine provinces [2-4]. In Salta and Jujuy provinces, malaria transmission historically started in October or November [5,2]. At present, the transmission season, December to March, has been recorded only for Salta [6]. Approximately, 99% of the cases are caused by *Plasmodium vivax*, although previously, in the 20th century, *Plasmodium falciparum *and *Plasmodium malariae *were found as well [5]. Malaria transmission occurred mainly inside houses as a consequence of the high density, strong anthropophily and indoor resting tendency of *Anopheles *(*Anopheles*) *pseudopunctipennis *[2].

Recent studies on the behaviour of anopheline mosquitoes in Salta province showed a characteristic pattern of *An. pseudopunctipennis *being the predominant species with an peak abundance in the springtime. Maximum mean humidity was the climatic variable that best explained the abundance fluctuation of this species. Modifications in the natural environment for agricultural purposes, for instance, would imply the development of certain areas, the so-called "edges" that would favour an increase in the abundance of anopheline mosquitoes and the contact between the vector and the local inhabitants [6,7].

The present work is a continuation of a series of investigations on anopheline population dynamics and the first longitudinal study of malaria in north-western Argentina. Entomological findings were related with epidemiological data and both with climatic variables.

## Methods

### Study area

The study was carried out in the localities of El Oculto (23° 06' S, 64° 30' W) and Aguas Blancas (22° 43' S, 64° 22' W), located 65 and 2 km away from the Bolivian border, respectively. In El Oculto, rains have an irregular temporal and spatial regime, alternating dry and wet years. These are monsoon rains, where maximum rainfall coincides with maximum temperatures. Ninety percent of the total annual rainfall occurs between November and April, the wettest period being December-February. The mean annual rainfall is 734 mm, with a maximum in January (154 mm) and a minimum in June and August (4 mm). In Aguas Blancas, the weather is hot and humid, with a dry winter season and heavy summer rains. Mean annual temperature is 20°C, with a typical rainfall regime between 700 and 1800 mm on the slopes. Both localities are within the altitudinal floor of the piedmont of the subtropical mountainous forest. Tree species such as "horco quebracho" (*Schinopsis haenkeana*), "cebil moro" (*Anadenanthera macrocarpa*), "yellow timber" (*Phyllostylon rhamanoides*), "pink lapacho" (*Tabebuia avellanedae*) and "Salta cedar" (*Cedrela angustifolia*) grow in this mixed forest, which has no predominant species and has an under storey with numerous arboreal patches, bushes and ferns [8].

El Oculto has 73 inhabitants (32 adults and 41 children); available data are not discriminated by sex. Poor houses are built alongside the provincial highway. Facilities are few and there is no drinking water or electricity. Subsistence farming takes place on small plots of land where corn, sweet potatoes, cassava, avocados, mangos and bananas are cultivated; chicken and pigs are also reared.

In Aguas Blancas, there are 672 men and 731 women (available data are not discriminated by age class), living in brick houses or in adobe and straw huts with tin roofs. In neighbourhoods away from the village centre there are few facilities, while in the centre itself a few houses have electricity and drinking water. There is a Primary Health Care Centre where patients are treated and if necessary sent to the Oran hospital in San Ramón de la Nueva Orán city (23° 08'S; 64° 20' W) located at 51 km. The main economic activity is trade, carried out in the Bolivian border town of Bermejo. People from Aguas Blancas often cross the border either in boats called "chalanas", in hired cars or on foot. They cultivate corn and bananas in their kitchen gardens. Fishing along the banks of the Bermejo River is an important activity that engages not only the population of Aguas Blancas but also the inhabitants of the interior of the province.

### Meteorological data

Data were recorded from two different weather stations situated in Aguas Blancas (22° 43' 60" S, 64° 22' 00" W) and San Ramón de la Nueva Orán (23° 07' 60" S, 64° 19' 60" W), 23 km from El Oculto. Maximum and minimum values of temperature and humidity were recorded daily, and then the mean monthly temperature and humidity were calculated as well as their maximum and minimum mean values; rain is expressed as the monthly accumulated rainfall.

### Mosquito collection

Adult specimens were collected monthly, during two successive sunsets, using CDC light traps baited with CO_2 _in both localities during four rainy seasons, from December 2001 to November 2005. Traps were placed outside the houses between 100–150 m from each house, both in the forest itself and at the forest edges. The specimens were identified using the key of Wilkerson and Strickman [9].

### Parasitological and epidemiological data

Blood samples were taking from persons with malaria symptoms. Technicians from the National Coordination of Vector Control (National Health Ministry) examined giemsa-stained thick blood smears of each blood sample for parasite detection at 1,000× magnification. Positive samples were examined for identification of the *Plasmodium *species. All epidemiological information was filed, the main data being age, sex, address, occupation, previous history of malaria and the most recent movements of the individual before detection of the disease. All individuals suffering from malaria were immediately treated with primaquine and chloroquine for 14 consecutive days. At the same time, an active search was carried out to detect other possible positive cases in the locality of the initial malaria case(s) and focal sprayings with FENDONA^® ^6SC (concentrated solution at 6%) were conducted inside and around their houses. The main purpose was to cure affected individuals and to prevent the spreading of the disease by controlling the vector.

### Statistical analysis

Random Effect Poisson Regression [10] was used to analyse the effect of climatic variables on the abundance of both vector and malaria cases. This analysis was carried out with longitudinal data in which the resulting variable was repeatedly measured each month; in this way the observations obtained are not independent from each other.

To analyze the relationship between the malaria cases and the climatic variables in El Oculto, the model did not include random slopes but only the random intercept. This contrasts with Aguas Blancas, where the best model included random slopes.

The Incidence Rate Ratio (IRR) of each variable on the mosquito number and the number of malaria cases was also calculated. This index made possible the direct observation of the percentage of influence of each variable on the mosquito abundance and the incidence of malaria.

## Results

### Seasonal variation in *An. pseudopunctipennis *populations

*Anopheles pseudopunctipennis *was the predominant species in El Oculto (4224/6624 specimens) but not in Aguas Blancas (572/2477 specimens). *Anopheles *(*Nyssorhynchus*) *strodei*, *Anopheles *(*Nys*.) *argyritarsis *and *Anopheles *(*Nys*.) *rondoni *were also collected in both localities. Species belonging to other genera of mosquitoes, i.e., *Culex*, *Psorophora*, *Aedes*, *Ochlerotatus *and *Limatus *were also collected.

**Figure 1:**
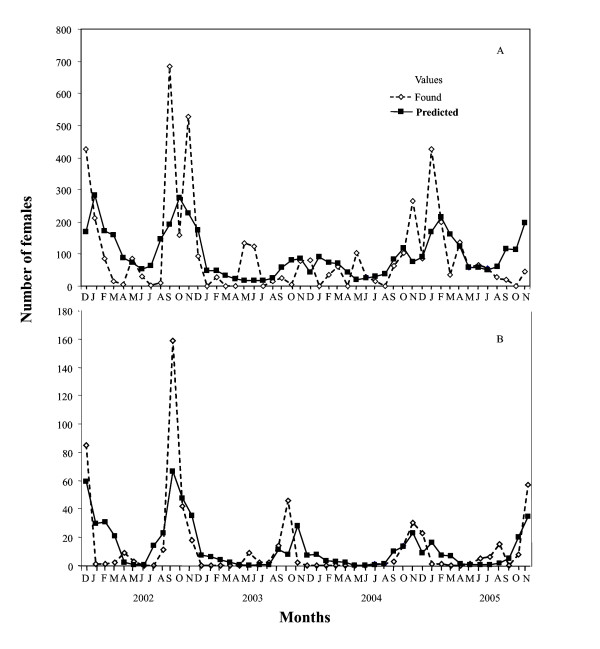
**Fluctuation in *Anopheles pseudopunctipennis *abundance based on the values obtained from the collections and the number of events predicted by the statistical model for (A) El Oculto, and (B) Aguas Blancas, December 2001 – November 2005**.

In El Oculto, *An. pseudopunctipennis *was characterized by three population peaks throughout the different sampling seasons, the largest during the spring season, the second during the autumn and the smallest during the summer season. During the winter, the number of mosquitoes dropped almost to zero. During the last year of sampling, a totally different pattern emerged, in which the number of specimens remained constant, not decreasing during the winter (Figure 1). In Aguas Blancas, there was a seasonal pattern throughout all the sampling years, with population peaks at the end of the spring season and at the beginning of summer (Figure 1).

In El Oculto, although the species exhibited a fluctuation without a marked seasonality, the model was able to predict such behaviour (Figure 1). In Aguas Blancas both observed and predicted values presented almost the same fluctuation (Figure 1).

### Relationship between the climatic variables and the population density of *An. pseudopunctipennis*

In El Oculto, the maximum mean temperature and the maximum mean humidity were the variables that best explained the fluctuation of the species (*P *< 0.001). According to the IRR, for each 1°C that the maximum mean temperature with respect to mean annual temperature increases, the density of the species increases 12%. Moreover, for each 1% that the maximum mean humidity with respect to the mean annual humidity increases, the mosquito population density decreases 1% (Table 1).

**Table 1:** Random Effect Poisson Regression for *Anopheles pseudopunctipennis *collected in El Oculto and Aguas Blancas, north-western Argentina

	El Oculto			Aguas Blancas		
	IRR	Standard Error	*P*	IRR	Standard Error	*P*

Intercept Maximum	73.79	56.025	0.001	4.652	3.880	0.049
Mean temperature Maximum	1.120	0.001	0.001	1.433	0.069	0.001
Mean humidity	0.976	0.005	0.001			
Mean humidity				0.960	0.008	0.001

First column indicates the climatic variables that were take into account in this study in El Oculto and Aguas Blancas localities; IRR: Incidence Rate Ratio indicates the influence of each variables in the locality, the Standard Error as well as the *P *value were calculated.

In Aguas Blancas, the maximum mean temperature and the mean humidity were the related variables. The IRR showed that for each 1°C that the maximum mean temperature with respect to mean annual temperature increases, the density of the species increases 43%, and with respect to mean humidity, for each 1% increase, the density decreases 1% (Table 1).

### Patterns of seasonal malaria transmission in relation to climatic variables

**Figure 2:**
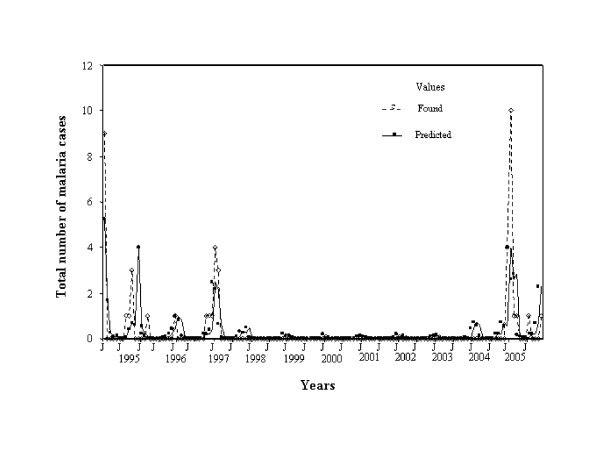
**Fluctuation in the number of malaria cases observed and predicted by the statistical model for El Oculto between January 1994 and January 2006**.

**Figure 3:**
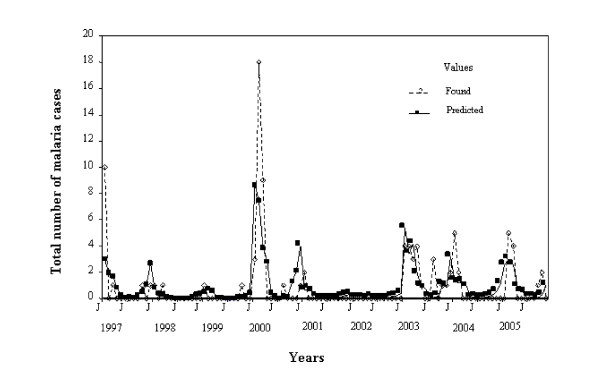
**Fluctuation in the number of malaria cases observed and predicted by the statistical model for Aguas Blancas January 1997 – December 2005**.

A total correspondence was observed for El Oculto between observed and predicted values of malaria cases despite a long absence of cases in the locality (Figure 2). For Aguas Blancas the predicted values also fitted the observed ones perfectly (Figure 3).

In El Oculto, the maximum mean temperature was the climatic variable that most significantly affected the disease fluctuation. For each 1°C increase in the maximum mean annual temperature, the risk of transmission of one case of malaria in the locality increases by 33%. Monthly-accumulated rainfall was also an important variable, since an increase in 1 mm in the mean annual rainfall increases by 0.1% the risk of the appearance of one case of malaria (Table 2).

**Table 2:** Random Effect Poisson Regression for malaria cases in El Oculto and Aguas Blancas, north-western Argentina

	El Oculto			Aguas Blancas		
	IRR	Standard Error	*P*	IRR	Standard Error	*P*

Intercept Maximum	0.003	0.070	0.001	0.132	0.257	0.015
Mean temperature	1.332	0.243	0.003			
Rainfall accumulated	1.009	0.003	0.001			
Mean temperature				1.592	0.400	0.004
Mean humidity				1.092	0.076	0.023

First column indicates the climatic variables that were take into account in this study in El Oculto and Aguas Blancas localities; IRR: Incidence Rate Ratio indicates the influence of each variables in the locality, the Standard Error as well as the *P *value were calculated.

In Aguas Blancas, the best fitting model included the mean temperature and humidity. Estimation using the model showed that if mean temperature and humidity are equal to their respective annual averages, the mean malaria risk rate is 0.13. If mean temperature increases 1°C with respect to the annual average, the incidence rate is expected to increase by 59%. If mean humidity increases 1% with respect to the annual average, the incidence rate of malaria increases by 1% (Table 2).

### Relation between malaria cases and vector abundance

**Figure 4:**
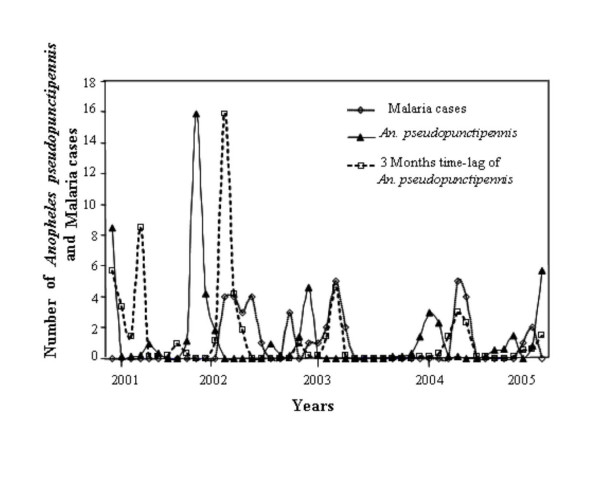
**Fluctuation in the number of malaria cases and *Anopheles pseudopunctipennis *specimens with a 3 months time-lag for Aguas Blancas between November 2001 and March 2006**.

On the basis of the values predicted by the model, a peak of mosquito abundance could be observed in Aguas Blancas and, after three months, a corresponding peak in the number of malaria cases (Figure 4). This relationship was not observed in El Oculto, since there were no cases for a long time that coincided with the abundance of mosquitoes registered for that period.

### Seasonal patterns of malaria cases

In El Oculto, 45 cases of malaria were registered during the 1994–2005 period, all identified as *P. vivax*. With respect to age groups, it is considered that the groups corresponding to < 20 years and > 50 years indicate the autochthonous transmission of the disease because this group spends more time inside houses, as opposed to young adults who work outside their homes. The migration related to work on farms or in commercial activities implies the movement of people to other localities with malaria transmission, where they acquired the disease (Vianconi, com. pers. National Coordination of Vector Control). 47% of the affected individuals were under 20 years of age, and 27% above 51 years (Table 3), therefore, these age groups were exposed in their houses to mosquito biting and closely connected with the autochthonous transmission of the disease.

**Table 3:** Incidence of malaria cases in El Oculto and Aguas Blancas localities, based on gender and age groups

Age groups (years)	El Oculto				Aguas Blancas			
	Male		Female		Male		Female	
		
	Number	%	Number	%	Number	%	Number	%

< 20 years	15	45.5	6	50.0	22	38.6	21	44.7
21–35 years	0	0.0	3	25.0	20	35.1	9	19.1
36–50 years	5	15.1	2	16.7	10	17.5	13	27.7
> 51 years	11	33.3	1	8.3	5	8.8	4	8.5
unkown	2	6.1	0	0	0	0	0	0
								
Total	33	100.0	12	100.0	57	100.0	47	100.0

In Aguas Blancas, 104 malaria cases were registered during the 1997–2004 period and all were *P. vivax*. The prevalence of the disease was greater by 41% in individuals under 20 years of age, but the young people involved in commercial activities (50%) were sick too. This locality, in front of Bermejo city (Bolivia), has a huge migration of people, and normally has not only autochthonous but also imported and introduced malaria cases (Table 3).

The percentage of cases involving young (< 20 years) and elderly (> 51 years) people was 74% and 50% for El Oculto and Aguas Blancas, respectively, with a high prevalence of cases in males (79%) for El Oculto in comparison with Aguas Blancas (47%). A great difference with respect to the origin of the cases was also observed, 12% and 100% being autochthonous for Aguas Blancas and El Oculto, respectively.

## Discussion

Epidemiologically, north-western Argentina was an important region in view of the series of malaria outbreaks that began at the end of the 19^th ^century, recording *P. falciparum*, *P*. *vivax *and *P*. *malariae *as aetiological agents [5]. The transmission season started in October or November in Salta and Jujuy provinces, in November or December in Tucumán, Santiago del Estero and Catamarca provinces, and in December and January in La Rioja and Córdoba provinces, being delayed if the previous winter or spring had been very cold or the spring intensely rainy. The transmission season could last until May or June for the whole area, longer during mild winters [5,2].

Entomologically, *An. pseudopunctipennis *was considered the main vector for the region. Its abundance was directly related to the subtropical climate and to the quantity of rainfall. Rains fed numerous permanent stream-waters, and the presence of suitable soils, favoured the growth of vegetation appropriate for the development of the vector, such as *Spirogyra majuscula *[5]. Thus, the years with normal rainfall caused high malaria morbidity. In contrast, very rainy or very dry years caused a lower morbidity, with streams washing away the breeding places, while during the latter their size was reduced, that is, the adult mosquito population decreased and so did the incidence of the disease. The manner and timing of the more intense rainfall played an important role. If it occurred before the anopheline reproductive season, the rivers and streams would become breeding sites, while if rainfall occurred during the reproductive season, rain would wash away the breeding sites, reducing malaria transmission [11,2].

In the present work, the influence of the climatic variables has determined differences in the patterns of behaviour of both the vector and the disease. *Anopheles pseudopunctipennis *seems to be extremely well adapted to the climate of El Oculto, since it presents not only a higher abundance than other species but also a more or less continuous seasonal distribution. The situation is different in Aguas Blancas, where the species is similar or lower in number compared with anophelines such as *Anopheles strodei *and *Anopheles argyritarsis*. A marked seasonality was also observed in the species, with peaks towards the end of spring and beginning of summer, when climatic conditions were mild.

Dantur Juri *et al *[6] reported that the fluctuation of *An. pseudopunctipennis *in El Oculto showed a marked seasonality with a peak during spring when environmental conditions favoured the breeding of the species, maximum mean humidity being the variable most highly correlated with abundance. In this work, the maximum mean temperature and maximum mean humidity influenced the fluctuation of *An. pseudopunctipennis*, not only in El Oculto but also in Aguas Blancas. It is not clear why in one locality a rise in temperature increased the density of the vector throughout the different seasons, while in the other it did so only for the spring-summer season. Perhaps the fact that in El Oculto the maximum mean humidity negatively influences the increase in mosquito abundance implies a certain relationship that has no simple explanation.

In Aguas Blancas, the greater abundance of the vector towards the end of spring and beginning of summer was related to the appearance of malaria cases in the summer and autumn. Malaria transmission in this locality showed a distinctive seasonality, unlike than in El Oculto, where the abundance of the vector could not be related to the cases of malaria due to the absence of cases for a long time.

Maximum mean temperature was the climate variable most related to the increase in malaria cases in El Oculto while in Aguas Blancas there was a closer relationship with mean temperature.

The fact that the rise in maximum mean temperature increases the density of the vector as well as the incidence of the disease could explain the absence of malaria cases throughout this prolonged period. It appears that a certain temperature is required to keep the parasite in the environment and for the parasite to be transmitted by the vector.

In Aguas Blancas, mean humidity was also significant and, on closer investigation, we noticed that in March 2002 there was a change in the annual average, which seems to have a bearing on the abundance and seasonality of the malaria cases.

In El Oculto, the statistical model predicted a series of malaria cases that were not found in nature, suggesting that the model needs further refinement or perhaps other interpretations. For example, the human population might have developed resistance following years of exposure to *P. vivax*, which could account for the low incidence of the disease among adults and the presence of certain asymptomatic cases. This situation was observed not only by Camargo *et al *[12] for a locality in Rondonia (Brazil), but also by Clyde [13]. The increase in anti-*Plasmodium *seropositive titres with age in the population in Rondonia [14] could be related to a certain amount of protection or attenuation of the *P. vivax *symptoms [15]. The relationship between age and immunity could favour the clinical manifestation of malaria especially in young people. In north-western Argentina, Mühlens *et al *[5] stated that more than half of the cases were of chronic malaria, with numerous parasites in the blood, but the absence of fever bouts meant they were not recognized as cases.

Finally, in the present study in both localities the infection was caused by *P. vivax *and individuals under 20 years of age presented a higher incidence. Although this last characteristic indicated a profile of autochthonous malaria, this situation can only be inferred for El Oculto, since in Aguas Blancas the constant population movement made it impossible to determine exactly where any given infection occurred. A lower number of cases in adults were found in Aguas Blancas, which would be related to a certain attenuation of the malaria symptoms.

The situation in these two localities of north-western Argentina indicates that in both there are certain conditions that favour the appearance of malaria cases. Further elaboration of these conditions would help to understand the cycle of the disease.

*Anopheles pseudopunctipennis *presented a typical fluctuation with major abundance in the spring in El Oculto, and the beginning of the summer in Aguas Blancas. Maximum mean temperature was the climatic variable most related to the species fluctuation in both localities. Malaria cases were influenced by maximum mean temperature and mean temperature in El Oculto and Aguas Blancas, respectively. The species fluctuation could be related to the presence of malaria cases in Aguas Blancas, where a three-month lag was observed. In El Oculto, this situation did no occur because there were no cases of malaria during a long time period.

It is important take into account that this situation involves local inhabitants. In El Oculto, a great number of autochthonous cases were found because the people did not go anywhere, reducing the possibility of acquiring the disease by migration. This does not happen in Aguas Blancas, where imported or introduced cases appeared too. For a better understanding of local conditions, further studies could be undertaken including migration processes and economical conditions that perhaps are influencing these ecological and epidemiological studies.

## Conclusion

The study of bioecological aspects of *An. pseudopunctipennis *was carried out mostly in México, North America. In South America research was focused on others malaria vectors such as *Anopheles darlingi*, *Anopheles albimanus *and *Anopheles aquasalis*. The present study is an important contribution to knowledge of the vector situation in the extreme of South America. Besides, the study of malaria cases was done in all the countries of America, but not focused on the relationship between the malaria cases and climatic variables in natural conditions. In this study, *An. pseudopunctipennis *fluctuation and malaria cases were affected by the maximum mean temperature. If this temperature increases, the population of the species and cases of malaria increases too. These new findings are the baseline for futures research that will be mainly focus on the relationship between the environmental conditions and the vectorial capacity of *An. pseudopunctipennis *in north-western of Argentina.

## Competing interests

The authors declare that they have no competing interests.

## Authors' contributions

MJDJ is a PhD student, this research is part of her thesis. She performed the survey (field sampling) and made the mosquito identifications. Besides, MJDJ collected and processed the information about malaria cases. Also MJDJ interpreted the data analysis and prepared the manuscript. MS performed the statistical analysis to this study. MZ, also collected mosquito material and as a part of Ministry of Health of the Argentina, gives the malaria information. GLC and WRA participated in the edition and in the critical reviewing of the manuscript. All the authors are in agree with this manuscript.

## References

[B1] World Health Organization & UNICEF (2005).

[B2] Bejarano JFR (1959). Áreas palúdicas de la República Argentina. Primeras Jornadas Entomoepidemiológicas Argentinas.

[B3] Bejarano JFR (1959). *Anopheles *de la República Argentina y sus relaciones con el paludismo. Primeras Jornadas Entomoepidemiológicas Argentinas.

[B4] Martínez-Palomo A (1991). Paludismo, de la euforia al desconcierto. Ciencia Hoy.

[B5] Mühlens P, Dios RL, Petrocchi J, Zuccarini JA (1925). Paludismo en el Norte Argentino. Rev Inst Bacteriológico del Dep Nac de Higiene.

[B6] Dantur Juri MJ, Zaidenberg M, Almirón WR (2003). Fluctuación estacional de *Anopheles (Anopheles) pseudopunctipennis *(Diptera: Culicidae) en un área palúdica de Salta, Argentina. Entomol y Vect.

[B7] Dantur Juri MJ, Zaidenberg M, Almirón WR (2005). Distribución espacial de *Anopheles (Anopheles) pseudopunctipennis *(Diptera: Culicidae) en un área palúdica de las Yungas de Salta, Argentina. Rev Saúde Pública.

[B8] Prado DE, Brown AD, Grau HR (1995). Selva pedemontana: contexto regional y lista florística de un ecosistema en peligro. Investigación, conservación y desarrollo en las selvas subtropicales de Montaña.

[B9] Wilkerson RC, Strickman D (1990). Illustrated key to the female Anophelinae mosquitoes of Central America and Mexico. J Amer Mosq Control Assoc.

[B10] StataCorp (2005). Stata Statistical Software: Release 9.

[B11] Bejarano JFR (1956). Distribución en altura del género *Anopheles *y del paludismo en la República Argentina. Revista Sanitaria Mil Argentina.

[B12] Camargo LMA, Noronha E, Villalobos Salcedo JM, Dutra AP, Krieger H, Pereira da Silva HL, Camargo EP (1999). The epidemiology of malaria in Rondonia (Western Amazon region, Brazil): study of a riverine population. Acta Trop.

[B13] Clyde DF (1989). Epidemiologic significance of immunity in vivax malaria. Epidemiol Rev.

[B14] Camargo LMA, dal Colleto GMD, Ferreira MU, Gurgel Sde M, Escobar AL, Marques AL, Marques A, Krieger H, Camargo EP, da Silva LH (1996). Hypoendemic malaria in Rondonia (Western Amazon Region, Brazil): seasonal variation and risk groups in an urban locality. Am J Trop Med Hyg.

[B15] Karunaweera ND, Carter R, Grau GE, Mendis KN (1998). Demonstration of anti-disease immunity to *Plasmodium vivax *malaria in Sri Lanka using a quantitative method to assess clinical disease. Am J Trop Med Hyg.

